# Role of Natural Products in Modulating Histone Deacetylases in Cancer

**DOI:** 10.3390/molecules24061047

**Published:** 2019-03-16

**Authors:** Myriam Merarchi, Gautam Sethi, Muthu K. Shanmugam, Lu Fan, Frank Arfuso, Kwang Seok Ahn

**Affiliations:** 1 Faculty of Pharmacy, University of Paris Descartes, 75006 Paris, France; myriammerarchi@hotmail.fr; 2 Department of Pharmacology, Yong Loo Lin School of Medicine, National University of Singapore, Singapore 117600, Singapore; phcsmk@nus.edu.sg (M.K.S.); phcfanl@nus.edu.sg (L.F.); 3 Stem Cell and Cancer Biology Laboratory, Curtin Health Innovation Research Institute, School of Biomedical Sciences, Curtin University, Perth, WA 6009, Australia; frank.arfuso@curtin.edu.au; 4 College of Korean Medicine, Kyung Hee University, 24 Kyungheedae-ro, Dongdaemun-gu, Seoul 02447, Korea; 5 Department of Korean Pathology, College of Korean Medicine, Kyung Hee University, 1 Hoegi-Dong Dongdaemun-Gu, Seoul 130-701, Korea

**Keywords:** HDACs, cancer, natural products, proliferation, metastasis

## Abstract

Histone deacetylases (HDACs) are enzymes that can control transcription by modifying chromatin conformation, molecular interactions between the DNA and the proteins as well as the histone tail, through the catalysis of the acetyl functional sites removal of proteins from the lysine residues. Also, HDACs have been implicated in the post transcriptional process through the regulation of the proteins acetylation, and it has been found that HDAC inhibitors (HDACi) constitute a promising class of pharmacological drugs to treat various chronic diseases, including cancer. Indeed, it has been demonstrated that in several cancers, elevated HDAC enzyme activities may be associated with aberrant proliferation, survival and metastasis. Hence, the discovery and development of novel HDACi from natural products, which are known to affect the activation of various oncogenic molecules, has attracted significant attention over the last decade. This review will briefly emphasize the potential of natural products in modifying HDAC activity and thereby attenuating initiation, progression and promotion of tumors.

## 1. Introduction

Histone deacetylases (HDACs) refers to a family of enzymes that remove the acetyl function from lysine amino acids in proteins and thus control many cellular and molecular processes. It has been found that HDAC inhibitors (HDACi) constitute a rising class of pharmacological drugs for the treatment of several chronic diseases, among which we can find cancer. Cancer is the second most common cause of mortality worldwide. However, after a generally positive response to initial therapy, many patients eventually develop recurrence as well as spread of the primary tumor, and it has been shown that aberrant HDAC enzyme activity can regulate tumor cell proliferation, survival, and metastasis. Several bioactive natural compounds have demonstrated their effects in treating and preventing cancer. Indeed, natural products constitute an abundant source for the discovery of anti-cancer drugs, and can modulate various important hallmarks of tumor cells. Almost 80% of all drugs approved by the United States Food and Drug Administration (FDA) over the last four decades for cancer’s treatment are either natural products or their derivatives. Hence, the discovery and development of novel HDACi from natural products has attracted significant attention over the last decade [1,2,3,4]. So far, few HDACi have received the FDA’s approval for the treatment of various cancers including, Vorinostat (SAHA, trade name; Zolinza^®^) [5], romidepsin (Istodax) [6], belinostat (Beleodaq) and panobinostat (Farydak) [7]. This review will briefly summarize the potential of few significant natural compounds in modifying HDAC activity, and thereby attenuating the initiation, progression, and promotion of tumorigenesis.

Among the molecular processes that HDACs regulate is modification of histone tails by the removal of the acetyl part from lysine amino acids, which will lead to the squeezing of the molecular interactions between the histones that have positive charges and the DNA that have negative charges, resulting into the opening of the chromatin structure to allow its binding to transcription factors to facilitate the transcription process. Thereby, when the HDACs deacetylate the histones, it leads to the strengthening of their interaction with DNA, and a closer chromatin structure, in addition to the inhibition of gene transcription [8,9,10,11,12]. Thus, HDACs have a main function in modulating epigenetic changes. HDACs can also control the post-translational acetylation of numerous non-histone proteins, such as different signaling molecules which can promote protein integrity changes, protein/protein or DNA interactions [13]. HDACs can also operate as regulators during post-translational modifications since they deacetylate non-histone proteins including important transcription factors, such as E2F, phosphoprotein p53, c-Myc, and nuclear factor-kappa B (NF-κB), Stat3, β-catenin etc, which can regulate cellular homeostasis [14]. Moreover, once non-histone proteins are deacetylated by HDACs it can result in their degradation by ubiquitination [15,16].

So far, it has been possible to identify and segregate 18 mammalian HDACs into four classes: class I HDACs (HDACs 1, 2, 3, and 8), class II HDACs (subdivided into two subgroups: class IIa, (HDACs 4, 5, 7, 9) and class IIb (HDACs 6 and 10), class III (sirtuin family: sirt1-sirt7), and class IV (HDAC 11) [17]. The analysis of their structures showed that class I, II, and IV HDACs have a common conserved domain for catalysis, with a similar catalytic core for acetyl-lysine hydrolysis that is Zn^2+^ -dependent, which led to the discovery and synthesis of new HDACi that occupy the catalytic core of the zinc-binding site, while class III HDACs require a nicotinamide adenine dinucleotide to have a catalytic activity; however, so far class III HDACs are not hindered by conventional HDACi [18,19,20,21]. 

Class I and Class II are distinguishable by different characteristics, including their location (nucleus for Class I, cytoplasm for Class II) after being phosphorylated by protein kinase C or D in the nucleus and shuttled to the cytoplasm. For the tissue localization in which they are expressed, Class I HDACs are ubiquitous, while the class II HDACs are synthesized specifically in particular tissues [22,23].

### 1.1. HDAC’s Role in Cancer

#### 1.1.1. Pro-Cancer Effects

Abnormal function of HDACs is related to main cancer key events. Enzymes linked with epigenetic regulation are frequently dysregulated in human cancers especially through mutation and abnormal expression [24]. In the case of the HDACs, several studies have indicated that there may be a direct correlation between their overexpression and significant reduction in both the recovery and survival of patients; also, when over expressed, poor patient prognosis can be predicted independent of the cancer type and tumor stage in various malignancies such as in prostate [25], colorectal [26], breast [27], lung [28,29], liver [30], and gastric cancers [31].

Interestingly it has been found that −402, −20, and +182 CDKN1A regions can display a significantly increased acetylation status, and these levels were positively correlated in gastric tumors [32]. Moreover, HDAC1 and 2 can also function to mitigate the response of the ATM pathway to DNA damage [33]. In addition, modifications in histone acetylation are suggested to drive the onset, proliferation, and metastasis of malignant tumors through the loss of monoacetylation and trimethylation on histone H4 [34,35]. Moreover, it has been demonstrated that the expression of HDAC1, −5, and −7 can act as a molecular biomarker to differentiate between malignant versus normal tissues [36]. 

#### 1.1.2. Anti-Cancer Effects

However, several reports have also demonstrated that the overexpression of HDAC cannot be always systematically attributed to a poor prognosis in cancer patients; indeed, in women with estrogen receptor positive (ER-positive) breast cancer, a decrease in HDAC6 levels was associated with a better prognosis [37]. Moreover, in cutaneous T cell lymphoma, an important clinical activity of pan-HDACis was observed, a correlation between an over expressed HDAC6 and an improved prognosis was noticed, while acetylated H4 was related with more aggressive lesions [38]. Overall, the various cancer types and pathways altered by HDACis are briefly summarized in Table 1.

### 1.2. HDACis from Natural Products

**Figure 1:**
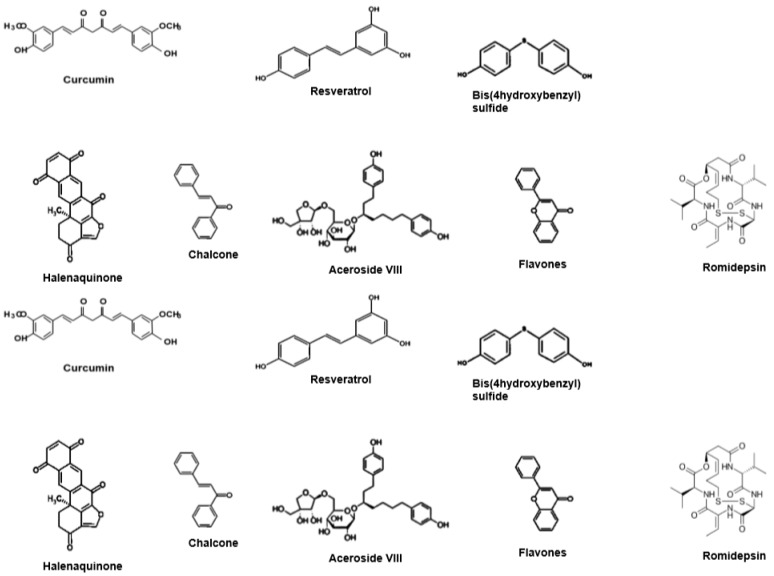
Structures of selected HDACi inhibitors from natural products.

A number of molecules derived from various medicinal plants have been reported to exhibit significnat antitumoral activities by affecting the activation/expression of various oncogenic molecules [2,3,4,39,40,41,42,43,44,45,46,47,48,49,50,51,52,53], including HDACs. Few such important natural compounds that can effectively alter the HDAC activity are briefly discussed below and their chemical structures are shown in Figure 1. 

#### 1.2.1. Resveratrol (RVT)

Resveratrol (RVT), a natural, biologically active polyphenolic compound that has exhibited its potential therapeutic application in the treatment of many diseases, including cancer [44,49,50,74,75,76,77,78]. The Nucleosome Remodeling and Deacetylase complex (NuRD) is one of the major chromatin remodeling complexes described to date in human cells and has an important role in the regulation of the transcriptional process. Metastasis associated protein 1 (MTA1) is a component of the NuRD that can induce gene silencing and is often upregulated in several cancers [79]. Phosphatase and tensin homolog deleted on chromosome 10 (*PTEN*), an anti-oncogenic gene, has been shown to be inactivated by MTA1 in malignant tumor cells [80].

In prostate cancer, RVT has been demonstrated to lead to the promotion of acetylation on Lys¹²⁵ and Lys¹²⁸ of PTEN as well as its reactivation by inhibiting of the MTA1/HDAC complex, which results in the inactivation of the PKB pathway. This suggests that the MTA1/HDAC complex regulates negatively the PTEN, and thus promotes proliferation as well as metastasis of prostate cancer, and that RVT can abrogate these key oncogenic hallmarks through the inhibition of MTA1 [54]. Another mode of action of RVT has been described through its activation of the class III HDACs. The docking studies clearly showed that RVT has a structure that could allow it to cause an inhibition of the function of various HDACs. 

In addition, in vitro analyses of global HDAC inhibition in human-derived hepatoblastoma cells has demonstrated that RVT can abrogate the expression/activity of 11 HDACs ranging from class I, II, and IV. In RVT treated hepatoma cell lines HepG2, Hep3B, and HuH7, an arrest of the proliferation of all cell lines in a concentration dependent manner has been noted. Interestingly, RVT caused a significant inhibition of HDACs, and hyperacetylation of the histones in HepG2 cells [55,56]. 

Interestingly, toxicity tests in primary human hepatocytes were positive as a good tolerance to RVT has been shown, while in vivo chicken embryotoxicity assays have shown toxicity at higher doses [56]. Moreover, it has been reported that combinatorial treatment with RVT and pterostilbene can cause the reactivation of ERα expression in ERα-negative MDA-MB-157 breast cancer cells, which is associated with an enrichment of acetyl-H3, acetyl-H3lysine9 (H3K9), and acetyl-H4 active chromatin markers in the ERα promoter region [57]. Panobinostat, an HDACi of the classes I, II, and IV Zn2+ catalytic domains demonstrates important anti-tumorigenic activities, specifically in lymphoid malignancies, ovarian cancer, and pancreatic cancer [81,82,83]. Sirtuin-1 (SIRT1), an NAD+ dependent class III HDAC, functions by deacetylating histones but also non-histone proteins and has a key function in cell survival and senescence but may not be modulated directly by HDACi, such as panobinostat. Once activated, SIRT1 can abrogate proliferation and induce apoptosis of lymphoid cells associated with deacetylation of STAT3 and NF-κB/p65, and repression of c-Myc protein levels. In malignant lymphoid cells, it has been also reported that panobinostat in combination with RVT can significantly enhance the pro-apoptotic effect of SIRT1 activators [84].

#### 1.2.2. Curcumin

Curcumin (diferuloylmethane) is a biphenolic active compound present turmeric, which is well known for its various pharmacological actions, against various chronic conditions including those in cancer [85,86,87,88,89,90,91], and is well established as a DNA methyltransferase inhibitor, and thus regarded as a DNA hypomethylating agent. It has been shown that curcumin can re-establishe the balance between HAT and HDAC 1, 3, 4, 5, 8 activity to specifically modulate the process of tumorigenesis [92]. Paired box gene1 (PAX1) has been characterized to be a tumor suppressor gene that is often hypermethylated and deactivated in various cancer types. Ubiquitin-like with PHD and RING Finger domains 1 (UHRF1) van function as a potent oncogene and may be hyperactivated in cancers [93,94]. The reactivation of PAX1 by curcumin was observed in HeLa (the first immortal cell line cultured by scientists), SiHa (grade II, human cervical tumor cell), and it has been suggested that the reactivation of PAX1 by curcumin may be attributed to its effect on histone deacetylase primarily caused through the downregulation of UHRF1, which may regulate both DNA methylation as well as histone acetylation [58]. 

Treatment of lymphoblastic Raji cells with curcumin was shown to decrease the amount of HDAC I and HDAC III through a proteasome-sensitive pathway. Those observations were combined with the evidence that curcumin can prevent degradation of the nuclear factor of kappa light polypeptide gene enhancer in B-cells inhibitor alpha (I κB α), an inhibitor of the pro-oncogenic NF-κB protein, and can also inhibit nuclear translocation of the NF-κB /p65 subunit, as well as the expression of the oncogenic Notch 1, and thus lead to the abrogation of cellular proliferation and tumor development [59]. Double-strand breaks (DSBs) are critical cytotoxic forms of DNA damage that can lead to genomic instability and then to tumor formation, if not properly repaired. Curcumin has been found to sensitize yeast cells to DNA-disrupting agents by the inhibition of Mec1 (ATR)-dependent pathway [60].

Cancer stem cells (CSCs) play an important part in the development of multi-resistant cancers including hepatocellular carcinoma (HCC). Among the main oncogenic pathways deregulated in liver CSCs, is that of NF-κB signaling. In HCC cells it has been shown that curcumin treatment led to a selective CSC-depletion and suppressed tumorigenicity, which was combined with substantial NF-κB inhibition. On the contrary, in curcumin-resistant cells, an augmentation in proliferation as well levels of CSC markers was noted. Moreover, co-administration of the class I/II HDACi trichostatin can also sensitize resistant cells to anti-neoplastic effects of curcumin [61].

In SkBr3 and 435eB cells treated with trichostatin A, addition of curcumin led to a more effective inhibition of cell growth, accompanied by a decrease in cells viability, and ERK, as well as Akt phosphorylation [63]. The combination treatment of curcumin with trichostatin A on SkBr3 cells led to cell cycle arrest and an increase in cyclin-dependent kinase inhibitor p21 and p27 expression, which is normally inactivated in most cancer [95]. There was also a decrease in cyclin D1 protein expression, whose overexpression has been demonstrated to be related to the evolution and progression of cancer [96]. Among the brain tumors, medulloblastoma is the most common one in children and young adults. In an in vivo medulloblastoma xenograft, curcumin significantly increased survival. This was associated with an induction of apoptosis and cell cycle arrest at the G2/M phase in medulloblastoma cells, in combination with a reduction in HDAC4 IV expression and activity, and an increase in tubulin acetylation [62]. Combined with the pan-HDACi, vorinostat and panobinostat, curcumin induced hyperacetylation of Hsp90 chaperone accompanied with a substantial depletion of Hsp90 client proteins (Akt, survivin, EGFR, and Raf-1), which caused both growth inhibition and apoptosis [64].

In human hepatoma cells, it has been reported that curcumin treatment can result in a potent inhibition of histone acetylation. Curcumin treatment led to a similar inhibition of histone acetylation in the absence or presence of trichostatin A. Also, the domain negative of p300 (a potent HAT protein) could block the inhibitory effect of curcumin on histone acetylation, and curcumin exposure reduced HAT activity. Furthermore, exposure of cells to low or high concentrations of curcumin reduced or enhanced reactive oxygen species (ROS) generation, significantly [65]. In the human pancreatic BxPC-3 cell line, it was observed that the treatment of EF24 (EF), a novel synthetic curcumin analogue, in conjunction with MS-275 (entinostat) or salermide (SAL) HDACi, can decrease the viability of BxPC-3 cells substantially. This effect was associated with an elevation in the acetylation of histone H3 and H4 as well as the number of cells in G1 phase, and a reduction in the ratio of cells in the G2/M phase [66].

#### 1.2.3. Marine Products

##### Actinomycetes Strains

In a recent study, led by Abdelfattah, numerous actinomycetes strains were isolated from various marine sponges collected from the Red Sea shore in Egypt to test the efficacy of their crude extracts as potential HDACi in the HeLa cell line. It has been demonstrated that the crude extract from *Streptomyces* sp. SP9 can exhibit a significant HDAC inhibitory activity. Heliomycin and tetracenomycin D the principal compounds of *Streptomyces* sp. SP9 and showed HDAC inhibitory activities and according to a computational docking study, the mechanisms by which tetracenomycin D may inhibit HDAC can be by promoting binding interactions with HDAC2 and HDAC3 [67].

Seidel C et al. isolated various Actinomycetes from 22 sediment samples along the Southern Coast of India that yielded 186 strains out of which 10 strains exhibited moderate to strong inhibition. The maximum inhibition (61%) was noted with strain VITKSM06 and the least (31%) with strain VITSJT03. The treated HeLa cells also showed a modified morphology and condensed chromatin, which may be attributed to HDAC inhibitory effects [68].

##### Marine Polycyclic Quinone-Type, Halenaquinone

In Molt 4 (lymphoblastic leukemia cells), K562 (chronic myelogenous leukemia cells), MDA-MB-231 (breast cancer cells), and DLD-1 (colorectal carcinoma cancer cells) the evidences have indicated that halenaquinone can induce apoptosis and inhibit proliferation, along with excessive production of ROS. Moreover, halenaquinone was found to reduce the activity of HDACs and the expression of the topoisomerase-IIα. Also the inhibition of the expression of the anti-apoptotic protein p-Akt was observed, in addition to the inhibition of some other important anti-apoptotic proteins including NF-κB, hexokinase II, and Bcl-2 upon halenaquinone treatment [69].

##### Other Products

Aceroside VIII: In HT29 human colon cancer cells, it has been demonstrated that Aceroside VIII, a diarylheptanoid isolated from *Betula platyphylla*, can selectively inhibit HDAC6 catalytic activity; and the combinatorial treatment of aceroside VIII with A452, a selective HDAC6 inhibitor, led to a synergistic elevation in the levels of acetylated α-tubulin leading to apoptosis and growth inhibition of the cancer cells [70].

bis(4-Hydroxybenzyl)sulfide: In the MDA-MB-231 breast tumor cell line, a sulfur containing compound, bis(4-hydroxybenzyl)sulfide (1), isolated from the root extracts of *Pleuropterus ciliinervis*, showed a potent inhibitory activity on the HDACs [71].

Chalcones: The HDAC inhibitory activity of various chalcones, natural phenols that form the central core for a variety of important biological compounds including *Leguminosae*, has been evaluated. It was demonstrated that isoliquiritigenin, butein, and homobutein caused an inhibition of both TNFα-stimulated NF-κB activation and HDAC activity [72].

Feijoa acetonic extract: Feijoa acetonic extract has exhibited tumor-targeting activities on various cancer cells. Flavone, its active component, was identified to induce apoptosis, in combination with a caspase activation as well as p16, p21, and TRAIL overexpression in leukemia cells. These effects were co-related to a higher histone and non-histone acetylation levels and by HDAC inhibition [73].

Romidepsin: The bicyclic peptide class-I selective HDACi romidepsin (Istodax) has been approved by the FDA as second-line therapy for both the treatment of cutaneous T-cell lymphoma (TCL) and of peripheral TCL. It acts as a prodrug, its disulfide bridge being reduced by glutathione upon entering into the cells, thus allowing the free thiol groups to interact with Zn ions in the active domains of class I HDACs. Romidepsin has been discovered during a program that aimed to evaluate the potential of fermentation products for their possible antimicrobial and antitumor activities. It can be naturally produced by *Chromobacterium violaceum*, is a large, motile, Gram-negative bacillus having a single polar flagellum and, usually, one or two lateral flagella. Presently, the commercial supply of romidepsin is being possibly generated by using a confidential fermentation process [97,98,99,100,101].

## 2. Conclusions

This review summarizes the anti-cancer actions of few important natural products against HDACs (Table 1). Accumulating evidence supports the paradigm that histone hypoacetylation and transcriptional dysfunction are involved in a number of cancer types. Several reports have clarified several unknown molecular and cellular targets implicated in HDAC inhibition as well as the mechanisms of action by which the anti-cancer activity can be achieved. These properties, combined with the good tolerance shown by their limited toxicity, suggest that HDACis derived from natural products deserve further fundamental animal and clinical studies in order to be used as novel therapeutics for the treatment of malignant tumors.

## Figures and Tables

**Table 1:** Modulation of HDAC activity by selected natural products in tumor cells.

	Cancer Types	Pathways/Molecules Altered	Concentration Range Tested	IC50	References
Resveratrol	Prostate cancer (DU145)	Akt↓, MTA1/HDAC I, II, IV complex↓, PTEN↑	5–100 µM	N.D.	[54]
	Hepatoma cancer (HepG2, Hep3B and HuH7)	HDAC I, II, IV↓	5–100 µM	32 µM (HepG2)	[55,56]
				200 µM (Hep3B)	
				29 µM (HuH7)	
Resveratrol + pterostilbene	Breast cancer (MDA-MB-157)	ERα↑, acetyl-H3↑, acetyl-H3 lysine9↑, acetyl-H4↑, HDAC↓, DNMT↓	15µM resveratrol + 5µM pterostilbene	N.D.	[57]
Curcumin	HeLa cells	PAX1 ↑	N.D.	N.D.	[58]
	SiHa cells	UHRF1 ↓			
Curcumin	B-non-Hodgkin lymphoma cell line	HDAC I↓, HDAC III↓, Notch 1↓, IκBα↑, p300↓	3.125–50 µM	25 µM	[59]
	*RPD3* mutants of yeast cells	HDAC↑, Mec1↓, Rad52↓, DSB repair↓	50–200 µM	N.D.	[60]
	Hepatocellular carcinoma	HDAC I/II↑ NF-κB↓		N.D.	[61]
	desmoplastic cerebellar medulloblastoma /DAOY tumor xenografts and Smo/Smo mice	HDAC VI↓ G2/M ↓ cleavage of caspase-3↑ tubulin acetylation↑	10–40 µM	N.D.	[62]
Curcumin + Trichostatin	Breast cancer (SkBr3 and 435eB)	HDAC I/II↓ pERK↓ pAkt↓ p21 and p27↑ p53↓ Cyclin D1↓ cleavage of caspase-3↑	10–20µM	N.D.	[63]
Curcumin + vorinostat/panobinostat		Hsp90 acetylation↑ EGFR↓ Raf-1↓ Akt↓ survivin↓			[64]
Curcumin + Trichostatin A	Human hepatoma	histone acetylation↓ HAT protein↓ ROS↑			[65]
EF24 + Entinostat or Salermide	Human pancreatic cancer (BxPC-3)	acetylation of histone H3 and H4↑ cells in G1 phase↑			[66]
Heliomycin	Cervical cancer (HeLa)	HDAC III↓		29.8 µM	[67]
Tetracenomycin D	Cervical cancer (HeLa)	HDAC II↓		10.9 µM	[67]
Nocardiopsis sp	Cervical cancer (HeLa)	HDAC↓		5.9 µM	[68]
Streptomyces sp	Cervical cancer (HeLa)	HDAC↓		7.2 µM	[68]
Halenaquinone	Lymphoblastic leukemia (Molt 4)	Oxidative Stress↑ Bax↑ PARP cleavage↑ caspase activation↑ cytochrome c↑ HDAC↓ Topoisomerase I & II↓		0.18 µM	[69]
	Human chronic myelogenous leukemia (K562)	p-Akt↓ NF-κB↓ HDAC↓ Bcl-2↓ hexokinase II↓		0.48 µM	[69]
	Breast adenocarcinoma (MDA-MB-231)	p-PTEN↓ p-GSK3β↓ p-PDK1↓ ROS↑		8 µM	[69]
	Colon adenocarcinoma (DLD-1)			6.76 µM	[69]
Aceroside VIII	Colon cancer (HT29)	HDAC VI ↓			[70]
Aceroside VIII + A452	Colon cancer (HT29)	HDAC VI↓ acetylated α-tubulin↑			[70]
Bis (4-hydroxybenzyl)sulfide (1)	Breast cancer (MDA-MB-231)	HDACs↓		1.45 µM	[71]
	Prostate cancer (PC3)	HDACs↓		7.86 µM	[72]
Chalcones: Butein	Human	HDACs I, II, and IV↓ TNFα↓ NF-κB↓	0–1000 µM	60 µM	[72]
	Philadelphia				
	chromosome				
	positive chronic myelogenous				
	leukemia				
	(K562)				
Flavone	Human myeloid leukemia	HDAC↓ caspase↑ p16↑ p21↑ TRAIL↑			[73]

*Abbreviations*: pAkt: Phosphorylated Protein kinase B. Bcl-2: B-cell lymphoma 2. Bax: Bcl-2-associated X protein. c-Myc: proto-oncogene. DSBs: Double-Strand DNA Breaks. ERα: Estrogen receptor-α acetyl-H3. EGFR: Epidermal Growth Factor. HAT protein: Histone acetyltransferase. DNMT: DNA methyltransferase. HDAC: Histone deacetylases. Hsp90: chaperone.IkB α: Nuclear factor of kappa light polypeptide gene enhancer in B-cells inhibitor alpha (I kappaB alpha). MTA1: Metastasis associated protein 1. Mec1: Serine/threonine-protein kinase. NF-*κ*B: transcription factor inhibitor. Notch 1: Notch homolog 1, translocation-associated (Drosophila). p300: Histone acetyltransferase. PAX1: Paired box gene1. PARP: Poly ADP ribose polymerase. p27 & p21: Cyclin-dependent kinase inhibitor. pERK: phosphorylated Extracellular signal-regulated kinases. PTEN: Phosphatase and tensin homolog deleted on chromosome 10. Raf-1: proto-oncogene serine/threonine-protein kinase. ROS: Reactive oxygen species. STAT3: Signal transducer and activator of transcription 3. SIRT: Sirtuin. TNFα: Tumor necrosis factor. TRAIL: TNF-related apoptosis-inducing ligand. UHRF1: Ubiquitin-like with PHD and RING Finger domains 1. ↑: Upregulation. ↓: Downregulation.

## Abbreviations

pAkt	Phosphorylated Protein kinase B
Bax	Bcl-2-associated X protein
c-Myc	Proto-oncogene
DSBs	Double-Strand DNA Breaks
ERα	Estrogen receptor-α acetyl-H3.
EGFR	Epidermal Growth Factor
HAT protein	Histone acetyltransferase
HDAC	Histone deacetylases
Hsp90	Chaperone
I κBα	Nuclear factor of κ light polypeptide gene enhancer in B-cells inhibitor α (I κB α)
MTA1	Metastasis associated protein
Mec1	Serine/threonine-protein kinase
Notch 1	Notch homolog 1, translocation-associated (Drosophila)
PAX1	Paired box gene1
PARP	Poly ADP ribose polymerase
p27 & p21	Cyclin-dependent kinase inhibitor
pERK	phosphorylated Extracellular signal-regulated kinases
PTEN	Phosphatase and tensin homolog deleted on chromosome 10
Raf-1	Proto-oncogene serine/threonine-protein kinase
ROS	Reactive oxygen species. STAT3: Signal transducer and activator of transcription 3
SIRT	Sirtuin
TNFα	Tumor necrosis factor
TRAIL	TNF-related apoptosis-inducing ligand
UHRF1	Ubiquitin-like with PHD and RING Finger domains 1.
